# The Mechanism-Based Inactivation of CYP3A4 by Ritonavir: What Mechanism?

**DOI:** 10.3390/ijms23179866

**Published:** 2022-08-30

**Authors:** Nancy H. C. Loos, Jos H. Beijnen, Alfred H. Schinkel

**Affiliations:** 1 The Netherlands Cancer Institute, Division of Pharmacology, 1066 CX Amsterdam, The Netherlands; 2 Faculty of Science, Department of Pharmaceutical Sciences, Division of Pharmacoepidemiology and Clinical Pharmacology, Utrecht University, 3584 CS Utrecht, The Netherlands; 3 The Netherlands Cancer Institute, Division of Pharmacy and Pharmacology, 1066 CX Amsterdam, The Netherlands

**Keywords:** ritonavir, CYP3A, mechanism of action, mechanism-based inhibitor/inactivator

## Abstract

Ritonavir is the most potent cytochrome P450 (CYP) 3A4 inhibitor in clinical use and is often applied as a booster for drugs with low oral bioavailability due to CYP3A4-mediated biotransformation, as in the treatment of HIV (e.g., lopinavir/ritonavir) and more recently COVID-19 (Paxlovid or nirmatrelvir/ritonavir). Despite its clinical importance, the exact mechanism of ritonavir-mediated CYP3A4 inactivation is still not fully understood. Nonetheless, ritonavir is clearly a potent mechanism-based inactivator, which irreversibly blocks CYP3A4. Here, we discuss four fundamentally different mechanisms proposed for this irreversible inactivation/inhibition, namely the (I) formation of a metabolic-intermediate complex (MIC), tightly coordinating to the heme group; (II) strong ligation of unmodified ritonavir to the heme iron; (III) heme destruction; and (IV) covalent attachment of a reactive ritonavir intermediate to the CYP3A4 apoprotein. Ritonavir further appears to inactivate CYP3A4 and CYP3A5 with similar potency, which is important since ritonavir is applied in patients of all ethnicities. Although it is currently not possible to conclude what the primary mechanism of action in vivo is, it is unlikely that any of the proposed mechanisms are fundamentally wrong. We, therefore, propose that ritonavir markedly inactivates CYP3A through a mixed set of mechanisms. This functional redundancy may well contribute to its overall inhibitory efficacy.

## 1. Introduction

### 1.1. Properties of Cytochrome P450 Enzymes including CYP3A4 and CYP3A5

Cytochrome P450 (CYP) enzymes form an essential superfamily of heme-containing monooxygenases that play an important role in the metabolism of countless endogenous and xenobiotic substances, including hormones, bile acids, and sterols, as well as many drugs [1,2]. CYPs catalyze hydroxylation and oxidation reactions, generally leading to the higher hydrophilicity of their substrates. In the case of drugs, this can sometimes result in prodrug activation but more often results in the formation of molecular structures that are more suitable for further conjugation in the body such as glucuronidation, sulfation, or glutathionylation [1]. These comparatively hydrophilic conjugates can generally be cleared more easily from the body than the parent drugs through excretion by the liver, small intestine, and kidneys. Although the liver is the major site of CYP-dependent drug metabolism, there are also variable levels of CYP expression in extrahepatic tissues, especially the small intestine, but also kidney, lung, and brain [3,4,5,6]. Furthermore, CYP expression can occur in several tumor tissues [7].

There are 57 CYP enzymes identified in humans, which are classified into 18 families, many of which have quite specific physiological functions [8]. Of most relevance for drug detoxification are the human CYP1, CYP2, and CYP3 families, which metabolize approximately 80% of all clinically used drugs. In this review, we focus on CYP3A, which is responsible for the primary metabolism of about 50% of drugs due to its extremely broad substrate specificity [1,8,9]. The most important isoform of this subfamily is CYP3A4, which is abundant in the adult liver and small intestine and is responsible for the bulk of the drug-metabolic activities of the CYP3A subfamily [1,10]. CYP3A4 activity is, therefore, an important determinant of the oral availability and overall systemic exposure of many of its substrate drugs. Since CYP3A4 activity can vary dramatically between but also within individuals due to its extensive inhibition or induction by a range of different drugs and food compounds, as well as by some genetic polymorphisms, this enzyme activity is a major cause of variable drug exposure in patients. This can lead to unpredictable underexposure (and thus under-treatment) but also lethal overexposure to a range of drugs [1,11].

Another important member of this subfamily, CYP3A5, has about an 83% amino acid identity with CYP3A4 and demonstrates extensive overlapping but not identical substrate specificities [12]. Judging from the 3D structures, most differences between the proteins are located in the helical F through G regions that form the flexible roof of the active site cavity and in the N-terminal region of the catalytic domain that shapes one side of the active pocket of CYP3A4 [13]. Consequently, the two enzymes have different active site structures: the active site of CYP3A5 is somewhat taller and narrower than that of CYP3A4 [13]. Some drugs are preferentially metabolized by either CYP3A4 or CYP3A5, and the exact nature or preponderance of the metabolites formed from shared substrates can differ between the two enzymes. CYP3A5 is relatively abundant in the kidneys and lungs and is polymorphically expressed in the adult liver and intestine [1,10]. CYP3A5 expression and activity levels are highly dependent on the genotype and ethnicity of an individual; although lowly expressed in most Caucasians (primarily carrying the poorly splicing CYP3A5*3 allele), it is highly expressed in most sub-Saharan African ethnic groups (primarily carrying the CYP3A5*1 allele). Many Asian ethnic groups have intermediate frequencies of the CYP3A5*1 allele and thus functional CYP3A5 activity [1,13,14,15]. For drugs that are preferentially metabolized by CYP3A5 over CYP3A4, the CYP3A5*1 genotype can, therefore, have an important impact on effective exposure to these drugs.

The monooxygenase reactions mediated by CYP3A4 and CYP3A5 are dependent on the interactions of the substrates with the heme iron atom. Generally speaking, these CYPs modify their substrates by using electrons received from NADPH through cytochrome P450 oxidoreductase (CPR) and/or cytochrome b5 (Cytb5) to reduce O_2_ to H_2_O and simultaneously generate an (often) hydroxylated or otherwise modified substrate [16,17,18]. The initial binding of substrates and (most) inhibitors to CYPs displaces a water molecule coordinated to the heme iron and alters the absorption spectrum of the heme group. Depending on the nature of the substrate binding, it can induce a type I or type II bound/unbound difference absorption spectrum. Substrates and inhibitors binding directly to the heme iron generally induce type II difference spectra, usually scanned in a wavelength range between 410 and 550 nm. When reducing equivalents (electrons) are available, hydroxylation or modification of the bound substrate by CYP3A can proceed, consuming oxygen (O_2_) and releasing water (H_2_O) in the process [18]. Spectrophotometric analysis of the heme group of CYP3As, especially around 450 nm, plays an important role in understanding their interaction with the substrates and will be frequently referred to in this review.

A striking feature of both CYP3A4 and CYP3A5 is their extremely broad substrate specificity, accommodating drug-like molecules with widely divergent structures and physicochemical properties, although many tend to be fairly hydrophobic. X-ray crystal structure analysis has revealed that this can be achieved because both enzymes have a large and flexible drug binding site. These binding sites can accommodate highly divergent substrate molecules, primarily using hydrophobic interactions. This, combined with the possibility of the induced fit of both the substrate molecule and the flexible peptide helices forming part of the drug binding site, can explain the extremely broad spectrum of drugs that are bound and metabolized by these proteins [13,14].

One important consequence of the broad substrate specificity of CYP3A4 and -3A5, with the drug binding sites accommodating many different molecules, is that these enzymes are also susceptible to reversible and irreversible inhibition by a large range of coadministered drugs or food compounds. Unsurprisingly, often irreversible inhibition (or inactivation) has the largest impact. For the broader group of CYP enzymes, four main mechanisms of inactivation have been described: the (quasi-irreversible) coordination of the substrate to the prosthetic heme iron by the formation of a metabolic intermediate complex (MIC); the covalent binding of a formed reactive intermediate to the apoprotein of the enzyme; the direct alkylation of a reactive intermediate to the heme prosthetic group; or the destruction of the prosthetic heme group resulting in heme-derived fragments that could covalently alter the apoprotein of the enzyme [11,19,20]. For some CYPs and inactivators, multiple mechanisms appear to apply side by side.

### 1.2. Ritonavir as a Clinically Important CYP3A Inhibitor

From a clinical perspective, CYP3A inhibition can be highly problematic, for example, it can unexpectedly enhance the exposure to a substrate drug, causing toxicity. However, inhibition can also be beneficial as quite a few drugs are so rapidly degraded by CYP3As that they do not reach or maintain therapeutic plasma levels. In such cases, CYP3A can be deliberately inhibited by the coadministration of a CYP3A inhibitor drug, for example, in HIV protease inhibitor booster regimens. Here, low-dose ritonavir (originally named ABT-538), which was originally developed as an HIV protease inhibitor, is often coadministered for its highly efficient CYP3A-inhibiting capacity [11,21,22,23,24]. In the following paragraphs, we provide some further background on the development and use of ritonavir in view of its importance as a clinical CYP3A inhibitor. During the early development of ritonavir as an HIV protease inhibitor, several issues were encountered. Ritonavir had a poor systemic availability, mainly due to the high CYP3A-mediated first-pass effects, and additional efflux by P-glycoprotein (P-gp/ABCB1) to the bile and intestinal lumen [24,25,26]. This poor oral availability required relatively high oral ritonavir doses of more than 1000 mg per day, resulting in a higher susceptibility to drug side effects, especially gastrointestinal adverse events [27]. Moreover, this high dose of ritonavir led to the concurrent induction of several CYP enzymes, including CYP3A4, -1A2, -2B6, -2C9, and -2C19, and also the induction of drug transporters, such as P-gp and the breast cancer resistance protein (BCRP/ABCG2), and uridine diphosphate-glucuronosyltransferase (UGT) [28,29,30,31,32]. This induction capacity of ritonavir is linked to the PXR and/or CAR signaling pathways [30,33]. However, even during chronic ritonavir administration, its potent CYP3A inhibitory effect appears to predominate [34].

The inhibitory capacity of ritonavir for CYP3A was first described by Eagling et al. (1997) and Von Moltke et al. (1998), reporting low in vitro K_i_ and IC_50_ values of 0.019 µM and 0.034 µM, respectively, based on the inhibition of testosterone 6β-hydroxylation [35,36]. As a result, it was realized that lower ritonavir doses could be used for its clinical application as a CYP3A inhibitor, namely 100–200 mg once or twice daily. These much lower doses reduced ritonavir’s side effects and caused much less enzyme induction, leading to markedly improved tolerability [37]. Another complication of using ritonavir as a single protease inhibitor (as for other HIV protease inhibitors) was the rapid development of viral resistance and subsequent reduced therapeutic efficacy. As a consequence, modern combination HIV regimens were developed, often including at least one protease inhibitor as well as an NNRTI [38].

A second-generation protease inhibitor, lopinavir (ABT-378), was developed to overcome some of the problems observed with ritonavir. Interestingly, lopinavir appeared to be rapidly metabolized by CYP3A and it was found that its oral availability could be dramatically boosted by the addition of ritonavir as a CYP3A inhibitor [39,40]. The combination of both protease inhibitors, first described by Sham et al. (1998), led to the development of the first fixed-dose combination of ritonavir and lopinavir (Kaletra) [41]. The use of ritonavir as a pharmacokinetic enhancer was further described in saquinavir drug–drug interaction studies [42]. More recently, this same principle was applied for the approved oral COVID-19 drug Paxlovid, containing the SARS-CoV2-targeting protease inhibitor nirmatrelvir (PF-07321332), which is rapidly degraded by CYP3A. This drug is co-formulated with a low dose of ritonavir in order to slow down its CYP3A-mediated degradation and thus extend its half-life and therapeutic efficacy [43,44,45].

**Figure 1:**
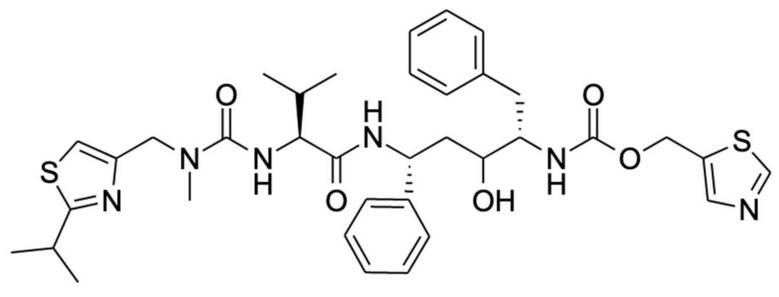
Chemical structure of ritonavir.

Ritonavir (Figure 1) is one of the most efficacious inhibitors of CYP3A4/5 in routine clinical use and is often used to deliberately boost the oral availability of drugs that are otherwise extensively metabolized by CYP3A4/5 [11]. However, it is also an important clinical investigative and research tool to establish which (especially newly developed) drugs are likely to be extensively affected by CYP3A4/5-mediated metabolism [46,47]. An important aspect of the clinical inhibition of CYP3A by ritonavir is its essentially irreversible nature in contrast with the efficient but fully reversible inhibition by compounds such as ketoconazole. Despite the reversible inhibitive nature of ketoconazole, the clinical CYP3A inactivation capacity of ritonavir appears only modestly higher in contrast with the inhibitory potency of itraconazole, which is significantly lower than that of ritonavir [48]. As a consequence of the irreversible action, once CYP3A is inhibited by ritonavir in vivo, it will remain nonfunctional, and only its replacement with newly synthesized CYP3A will lead to a recurrence of CYP3A activity. The duration of the inhibition should thus to a large extent be dependent on the CYP3A turnover rate in the tissues in question, which might be quite rapid, especially in the small intestine, where entire human enterocytes have a turnover period of only about 3.5 days [49]. Interestingly, there is no consensus in the literature about the clinical recovery time of CYP3A activity after the discontinuation of ritonavir. For example, a study by Culm-Merdek et al. (2006) described nearly full recovery after a three-day washout period, whereas Katzenmaier et al. (2011) observed the inactivity of the enzyme even after three days [50,51]. The former results would appear to be more in line with the known turnover rate of enterocytes, but the replacement of CYP3A in hepatocytes could be considerably slower.

Another important aspect of the long-term clinical use of ritonavir is that it does not seem to lose its boosting efficacy over time. Patients receiving ritonavir have sustained boosting effects despite extended periods of treatment, with little clinical evidence of induction of other CYP or UGT pathways [52].

It is worth noting that although the time-dependent inhibition of CYP3A by ritonavir, that is, the mechanism-based inactivation, was first described in the late 1990s, it is now known that ritonavir also exhibits a significant reversible inhibitory capacity [25,53,54]. As discussed somewhat later in this review, ritonavir thus exhibits a mixed CYP3A inactivation potency [55].

### 1.3. The Ritonavir Analogue Cobicistat Has Very Similar CYP3A Inhibition Properties

Interestingly, a close structural ritonavir analogue, cobicistat, is also registered (in 2014) and similar to other analogues is under further investigation for use as a clinical booster [56,57,58,59]. Similar to ritonavir, cobicistat is a highly potent CYP3A inhibitor with a reversible and time-dependent component. The CYP3A IC_50_ values of ritonavir and cobicistat are in the same range of 0.01–0.04 µM, although it seems that ritonavir has a slightly higher inhibitory potency compared to cobicistat. Nonetheless, the clinical effects as boosters are nearly identical for both ritonavir and cobicistat [55]. According to the manufacturer, cobicistat should be more selective than ritonavir. However, Hossain et al. showed that this drug can also inhibit other CYP enzymes, including CYP2B6, CYP2C19, and CYP2D6, to possibly an even greater extent than ritonavir [55]. In contrast, ritonavir also exhibits induction capacities through the PXR/CAR signaling pathways, whereas cobicistat appears to lack this ability to induce other CYP enzymes or transporters [60]. Therefore, cobicistat could present a better drug–drug interaction profile than ritonavir.

### 1.4. Aim of This Review

Surprisingly, despite its wide application, the exact mechanism of the irreversible inhibition of CYP3A by ritonavir is still only partly understood, and divergent hypotheses and models for its exact nature are currently being entertained by different research groups. This review aims to discuss these divergent models.

## 2. Binding of Ritonavir to CYP3A4 and CYP3A5

Ritonavir was originally developed based on its structure/activity relationship with respect to inhibiting HIV protease, its intended molecular target. Its strong interaction with CYP3A4 is purely coincidental and not based on the CYP3A4 crystal structure, which was still unknown at the time [56]. As discussed in this review, there are several theories about the primary mechanism of CYP3A inhibition or inactivation by ritonavir, which are not necessarily mutually exclusive. An important reason underlying the complexity in understanding this inhibition may be the flexibility and large size of the CYP3A drug binding site, allowing the induced fit of both the protein and/or its drug substrates and inhibitors. Moreover, multiple possible orientations and configurations of the substrates coordinating in the drug binding site, and even multiple substrates binding at the same time, can occur due to these properties [8,61,62].

**Figure 2:**
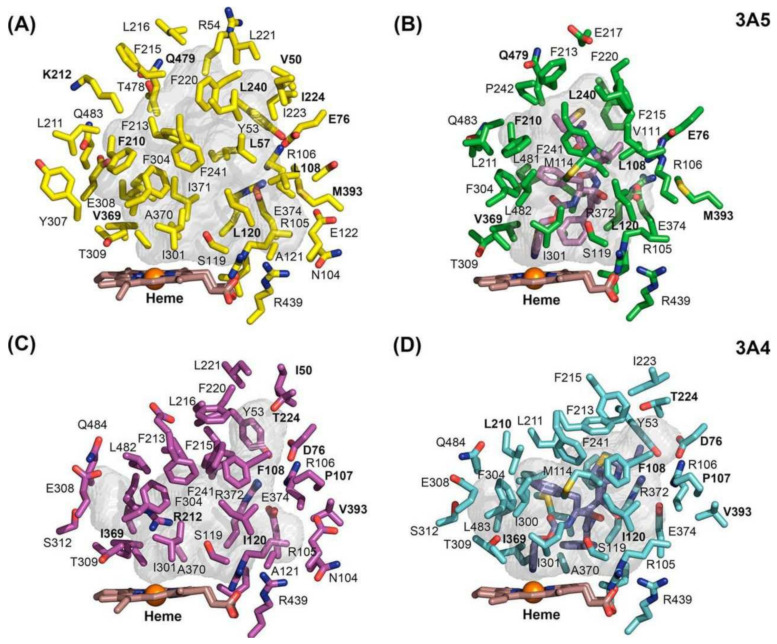
The active site cavities (grey shapes) of CYP3A5 and CYP3A4 in an unbound and ritonavir-bound state. (**A**) Active site cavity of CYP3A5 (amino acid (aa) side chains in yellow). (**B**) Active site cavity of CYP3A5 bound to ritonavir (aa side chains in green). (**C**) Active site cavity of CYP3A4 (aa side chains in magenta). (**D**) Active site cavity of CYP3A4 bound to ritonavir (aa side chains in cyan). Heteroatom colors are nitrogen (blue), oxygen (red), iron (orange), and sulfur (dark yellow). The active site of ritonavir-bound CYP3A5 is narrower and smaller in size compared to that of CYP3A4, which is adapting its width and height toward the ritonavir molecule. The thiazole group of ritonavir (pink purple in panel (**B**), blue purple in panel (**D**)) shows an equally tight association with the heme iron in both enzymes. This figure was reproduced with permission from [14].

As shown in a comparison of X-ray crystal structures by Hsu et al., ritonavir exhibits higher configurational entropy when bound to CYP3A5 compared to CYP3A4, which is associated with quite different shapes of the active site cavity (see Figure 2B,D) [13]. Still, the binding affinity of ritonavir for these two enzymes is similar even though ritonavir adopts partly different conformations when bound by CYP3A4 or CYP3A5. Importantly, in these X-ray structures, the thiazole group of ritonavir shows an equally tight association with the heme iron in both enzymes. This, in combination with the high hydrophobicity and relatively poor aqueous solubility of ritonavir, may explain the high binding affinity to CYP3A4 and -3A5 rather than a strict lock and key fit model [13,14]. The narrower active site of CYP3A5 leads to the redirection of the isopropyl-thiazole end of ritonavir to occupy an enlarged portion of the “roof” of the active site cavity above (and far away from) the heme [13,14]. The active site of CYP3A5 shows reduced plasticity compared to CYP3A4 due to the differences in the width and height of their active cavities (Figure 2). Ritonavir shows higher configurational entropy with CYP3A5 because the molecule has some degree of freedom inside the active site. In contrast, the active site cavity of CYP3A4 widens and increases in height in the structure of the CYP3A4-ritonavir complex [13]. This results in a highly restrained ritonavir molecule in this configuration of the CYP3A4 complex. Irrespective of the exact mechanism of binding and inhibition, experimentally it is clear that ritonavir is a potent (quasi-)irreversible inhibitor of both CYP3A4 and CYP3A5. However, due to the greater preponderance of CYP3A4 in the populations of most developed societies, ritonavir inhibition of CYP3A has so far been mostly investigated using CYP3A4.

## 3. Metabolism of Ritonavir by CYP3A4 and -3A5

Studies of the mechanism of inhibition of CYP3A by ritonavir are further complicated by the fact that CYP3A4 and CYP3A5 can also metabolize ritonavir to a range of different metabolites that are readily released from the proteins. Ritonavir is quite a complex, elongated molecule, containing 18 rotatable bonds allowing substantial structural flexibility (Figure 1). It may well be that different orientations or configurations of ritonavir during entering into or upon positioning inside the equally flexible active sites of these proteins can either result in productive metabolism or irreversible inhibition.

**Figure 3:**
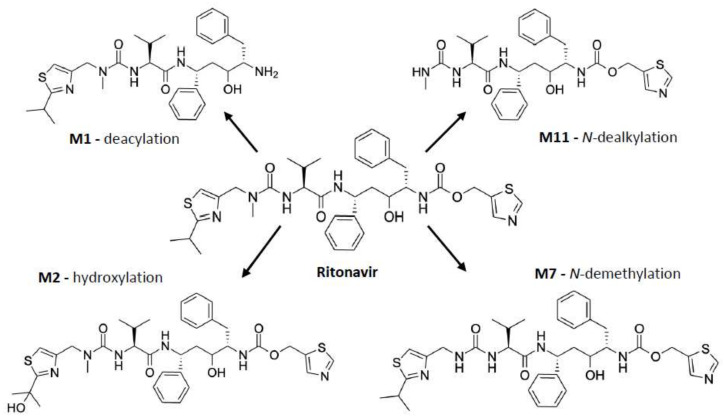
Ritonavir and its four major metabolites, which are M1 (deacylation), M2 (hydroxylation), and M11 (*N*-dealkylation) formed by CYP3A4 and/or CYP3A5; and M7 (*N*-demethylation) formed by CYP3A4/5, as well as CYP2D6 [63,66].

As shown in Figure 3, ritonavir is primarily metabolized by human CYP3A4/5 through *N*-demethylation, hydroxylation of the isopropyl side chain, and cleaving off of the terminal thiazole or isopropyl-thiazole groups, yielding four major metabolites amongst other metabolites formed during the biotransformation [61,63,64,65,66]. Of note, the *N*-demethylation reaction can also occur through human CYP2D6 [63]. The in vitro metabolism of ritonavir is characterized by an apparent *Km* of 0.1–0.5 µM for recombinant CYP3A4. Moreover, the reaction is nonlinear, with a progressive slowing down of the metabolic conversion, presumably caused by the developing irreversible inhibition of CYP3A4 [61].

## 4. Principal Mechanisms of Irreversible Inhibition of CYP Enzymes by Substrates

Many different CYP enzymes are known to be irreversibly inactivated by substrate-like drugs or drug-like molecules, and this can happen through different primary inhibitory mechanisms. For CYP3A4/5, given the complexity of their substrates and the multiplicity of the chemical modifications they can make on the same substrate, multiple mechanisms at the same time might be relevant even for one drug. For instance, upon metabolic activation, the substrate 17-alpha-ethynylestradiol can irreversibly inactivate CYP3A5 by both heme modification and covalent binding to the apoprotein [67]. The studies discussed in this review (Table 1) variously propose four fundamentally different, irreversible inhibition mechanisms of CYP3A4/5 by ritonavir, namely the (I) formation of a true metabolic intermediate complex (MIC); (II) extremely tight binding of unchanged ritonavir to the heme iron; (III) heme destruction; and (IV) formation of a covalent bond with the CYP3A polypeptide by a reactive intermediate of ritonavir. Some of these currently proposed inhibitory mechanisms of CYP3A4/5 by ritonavir are referred to as the mechanism-based inactivation of the CYP3A4/5 enzyme. These are time-dependent and roughly correspond to the four different described types of mechanism-based inactivation for CYPs in general [11,19,20]. Despite the differences between these proposed mechanisms of inactivation, the mechanism-based inactivators/inhibitors are generally considered to be compounds that are chemically converted by the target enzyme into a reactive intermediate or metabolic intermediate complex (MIC). This is then able to inactivate the enzyme by remaining attached to the active site or prior to its release from the active site cavity [20,53,61,68,69]. However, as we are interested in all of the effectively irreversible inhibition mechanisms of CYP3A, this definition may be too narrow. Therefore, in this review, we will consider all of the effectively irreversible CYP3A4/5 inactivation mechanisms described for ritonavir, even if they do not perfectly fit the above definition. For each of these proposed mechanisms of ritonavir-mediated inactivation, supporting experimental evidence has been provided as discussed below.

### 4.1. Inactivation of CYP3A by Formation of a Metabolic Intermediate Complex (MIC)

The formation of a metabolic intermediate complex (MIC) as a mechanism of CYP3A inactivation by ritonavir was first proposed by Ernest et al. (2005) [53]. The primary amine present in the metabolites of ritonavir (e.g., M1, Figure 3) may for instance be the structural moiety that could form a metabolic intermediate (MI). This MI (of unknown chemical structure) could then coordinate tightly with or chelate to the prosthetic heme in the active site of CYP3A, leading to a more stable (ferrous) state of the heme iron but without forming a covalent attachment with the polypeptide part of the protein [53]. This would be a true MIC. The extent of this type of MIC formation can be monitored by measuring the spectrophotometric peak absorbance shift in heme-associated light absorption at a wavelength of ~455 nm. Possible MIC formation was measured between ritonavir and insect cell microsomes containing recombinant CYP3A4 supplemented with Cyt*b*_5_ (CYP3A4(+*b*_5_)), recombinant CYP3A5 (not supplemented), or human liver microsomes (HLMs), all co-incubated with NADPH to regenerate (added) cytochrome P450 reductase (CPR). It is worth noting that Cyt*b*_5_ is naturally present in the in vivo cellular environment of CYP3A4, and it has been shown that the absence of Cyt*b*_5_ could affect the efficiency of CYP3A4 functioning [17,18]. Based on the absorbance shift, Ernest et al. observed apparent MIC formation of up to ~62% of the total CYP3A with CYP3A4(+*b*_5_) at 10 µM ritonavir, which increased with time over 60 min of incubation. However, neither CYP3A5 (not supplemented with Cyt*b*_5_) nor HLM yielded spectrophotometrically detectable MIC formation with ritonavir, although this might in part also be due to detection limitations [53].

These in vitro studies further showed a clear time- and concentration-dependent decrease of microsomal CYP3A metabolic activity upon incubation with ritonavir of all of the preparations, i.e., HLMs or insect-cell-expressed CYP3A4 and CYP3A5, with an IC_50_ of ~0.05 µM. This low IC_50_ of ritonavir would reflect high-affinity binding to CYP3A4, and the authors further assessed that there was a minimal nonspecific binding of ritonavir. More detailed kinetic analysis indicated that higher amounts of CYP3A4(+*b*_5_) protein required higher concentrations of ritonavir to obtain similar levels of inhibition, confirming that above 0.05 µM of ritonavir, substantial irreversible inhibition occurred [53]. The partition ratio of ritonavir is about 1, meaning that approximately 1 mole of the drug is required to inactivate 1 mole of the CYP3A4 enzyme [63]. However, below 0.05 of µM ritonavir, the reversible inhibition of CYP3A4(+*b*_5_) appeared to be more prominent, although the precise kinetic parameters were difficult to establish due to interference by the irreversible inhibition. Collectively, this study showed that there is substantial irreversible inhibition of CYP3A4 at concentrations above 0.05 µM of ritonavir. These findings suggested that mechanism-based inactivation plays an important role in the inhibition of CYP3A4 and CYP3A5 by ritonavir, although the inactivation appeared more rapidly for CYP3A4(+*b*_5_) than for CYP3A5 (not supplemented) [53]. We cannot exclude that the presence or absence of Cyt*b*_5_ may have affected these outcomes. However that may be, this type of inactivation of CYP3A would be consistent with the persistent inhibitory effect of ritonavir observed after the discontinuation of drug administration in the in vivo (clinical) setting.

### 4.2. Inactivation of CYP3A through Tight Binding to the Heme Iron

A second, very different inactivation mechanism of CYP3A by ritonavir was proposed by another research group (Sevrioukava and co-workers, in a series of studies starting in 2010). This group concluded that the essentially irreversible inhibitory effect of ritonavir was primarily due to the very tight binding of ritonavir to the heme iron. The concomitant marked decrease in the redox potential of the heme iron then virtually prevented its subsequent reduction by CPR [61]. Ritonavir is thought to remain unchanged during this interaction. These studies were mostly performed using CYP3A4Δ3-24, an engineered mutant protein lacking a 22-amino acid lipophilic membrane anchor that was removed in order to allow crystallization of the (now soluble) protein for X-ray structure determination. This modified protein had previously been demonstrated to catalyze mono-oxygenation reactions similar to the native protein. Of note, most ritonavir binding studies with this CYP3A4Δ3-24 protein were performed in the absence of CPR, NADPH, and Cyt*b*_5_.

Sevrioukova et al. (2010) showed that the spectral perturbations in the CYP3A4Δ3-24 heme upon the addition of ritonavir are indicative of the displacement of water as a sixth ligand from the heme iron and direct coordination of the heme iron to a nitrogen atom, suggesting a so-called type II ligand binding [61]. They concluded based on their spectral data that the thiazole nitrogen of ritonavir is most likely to ligand with the iron in both ferric (Fe^3+^) and ferrous (Fe^2+^) CYP3A4Δ3-24 [61]. The binding of ritonavir with the thiazole group to both CYP3A4 oxidation states was also found to be stoichiometric and effectively irreversible, and even large excesses (>100-fold) of type I CYP3A4 substrates (such as bromoergocryptine and progesterone) were readily displaced by the added ritonavir. Type I substrates tend to elevate the redox potential of CYP3A4 from about −330 mV to about −270 mV, facilitating reduction by CPR, whereas the type II substrate ritonavir was found to lower the redox potential to approximately −350 mV; this increased redox potential difference with CPR makes it thermodynamically highly unfavorable for CPR to reduce the ritonavir-bound CYP3A4, thus contributing to a virtually irreversible binding of ritonavir. Indeed, P450 turnover was inhibited by ritonavir, and ritonavir addition did not increase NADPH consumption in reconstituted CYP3A4 and CPR [61,70]. The authors, therefore, concluded that ritonavir is a very high-affinity type II ligand that inhibits CYP3A4 via strong ligation and lowering of the redox potential of the heme iron, thus preventing the acceptance of electrons from the redox partner CPR [61,70]. This essentially locks the protein in an inactive state.

An interesting question remains as to why the CYP3A4-ritonavir binding in these studies was found to be biphasic and why there was a big difference between the measured equilibrium and dissociation constants. This biphasic binding behavior could reflect the insertion of ritonavir into the active binding site in one (less stable) or another (more stable, irreversibly binding) orientation, or perhaps the slow rearrangement of the ritonavir structure inside the active site from the less stable to the irreversibly binding configuration. Sevrioukova et al. suggest that a possible explanation could lie in the elongated shape of the ritonavir molecule, which may thus enter the active site cavity of CYP3A4 in two different orientations, with either the thiazole or the isopropyl-thiazole end oriented towards the heme [61,70]. According to the authors, as the isopropyl-thiazole end group of ritonavir is readily hydroxylated by CYP3A4 (to M2, see Figure 3), this end group is unlikely to contain the inactivating heme iron-coordinating thiazole. Therefore, the opposite thiazole end group is most likely the one tightly coordinating with the iron atom to yield the inactivation of CYP3A [61]. Perhaps it is, therefore, simply a matter of which end of ritonavir enters the CYP3A4 binding site first that determines whether it gets metabolized or can irreversibly block the enzyme. Furthermore, it would mean that if ritonavir enters the active cavity of CYP3A4 with the isopropyl-thiazole head on, a positional/conformational rearrangement must take place within the active site cavity to orient the other thiazole moiety towards the heme iron [61]. Alternatively, this ritonavir could dissociate from CYP3A4 and may then reenter the active site cavity in the opposite orientation. Both of these processes would take time and can lead to a slow phase in the binding reaction, explaining the biphasic behavior [61]. A third reason for the biphasic binding behavior could relate to the conformational heterogeneity of CYP3A4, as its active site is structurally flexible and can adjust around ritonavir by adopting different conformations [61,70].

**Figure 4:**
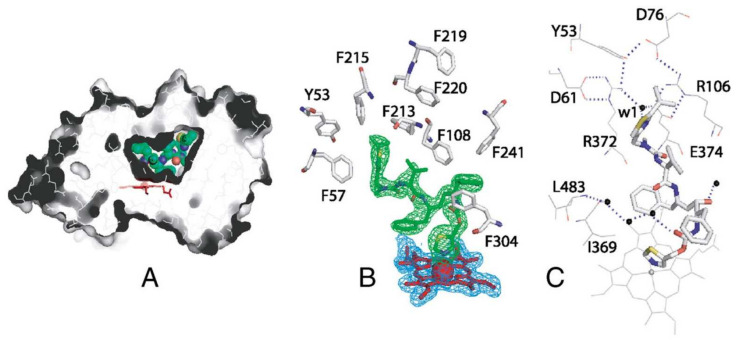
Crystal structure of the complex between CYP3A4 and ritonavir. (**A**) The active site cavity of ritonavir-bound CYP3A4, in which ritonavir is depicted in green and the heme in red. This illustrates that ritonavir is totally sequestered from surrounding water. (**B**) Aromatic residues surrounding ritonavir supporting the hydrophobic interactions inside the active site. Blue and green indicate the electron density around ritonavir and the heme. (**C**) An umbrella-like charge–charge/H-bonding network connected to the isopropyl-thiazole moiety of ritonavir via the highly ordered water molecule (w1), illustrating the electrostatic interactions with ritonavir. Reprinted with permission from [61], 2010, PNAS.

Sevrioukova and Poulos (2010, 2012) also established the X-ray crystal structure of a CYP3A4Δ3-24-ritonavir complex [61,70]. This indicated that ritonavir fits exceptionally well into the malleable active site cavity of CYP3A4, where it achieves extensive hydrophobic and charge–charge/H-bonding interactions with the protein through its side chains, yielding near-perfect complementary protein–ligand contacts. Ritonavir was also totally sequestered from the solvent (Figure 4) [70]. Moreover, in this crystal structure, the thiazole nitrogen was indeed found to be tightly ligated to the heme iron, with a short (2.2–2.3 Å) N-Fe distance, consistent with the spectral data. Thus, the X-ray structural data were in very good accordance with the biochemical results obtained by this group. In their interpretation, the binding of ritonavir to CYP3A4 is mainly irreversible due to the strong thiazole nitrogen coordination with the heme and near-perfect complementary protein–ligand contacts, as well as the resulting reduced redox potential of the heme iron [70].

Since ritonavir is a comparatively large molecule, it is unlikely that the active site of CYP3A4 can accommodate two or more ritonavir molecules. However, there is also the possibility that ritonavir is able to bind peripherally to CYP3A4 outside the active site cavity [70]. When this peripheral perhaps portal-like binding site of CYP3A4 is saturated, ritonavir translocates to the active site and interacts with the heme [70]. This peripheral binding of CYP3A4 could also influence the access and docking orientation of ritonavir to the active site [61]. Furthermore, the reorientation of ritonavir takes time and is possibly slow enough to enable CYP3A4 to receive two electrons from CPR while ritonavir is still in a different configuration. This could then lead to the formation of a reactive intermediate as reported by other groups (discussed below). Ritonavir is thus converted into a reactive intermediate upon oxidation and may then selectively inactivate CYP3A4 through an irreversible bond to the heme scaffold and/or active site amino acid residues. It was further suggested that the isopropyl-thiazole end group was involved in the formation of this putative reactive intermediate [61].

In a follow-up study, Sevrioukova et al. further demonstrated that the active site arginine Arg212 (not shown in Figure 4) of CYP3A4 may be important for the binding affinity and catalytic transformation of large substrate molecules such as ritonavir [70]. Arg212 belongs to one of the substrate recognition regions of the active site and could facilitate the ligation of the thiazole end of ritonavir to the heme. It seems that this specific amino acid is essential for the binding of CYP3A4 substrates and is involved in the catalytic transformation of ritonavir. However, its possible role in inactivating the enzyme was not specifically considered. The authors further do not exclude that there may be multiple different orientations of ritonavir bound to CYP3A4, some of which may allow the formation of reactive metabolites and intermediates [70]. In fact, the observation that structurally widely different ritonavir metabolites can be generated by CYP3A4 (Figure 3) already proves that various reactive configurations between ritonavir and CYP3A4 must exist.

In a more recent study involving ritonavir analogues (2018), this group likewise suggested that ritonavir-like compounds could also inactivate CYP3A4 via strong ligation to the heme iron. This leads to a blockade of the active site and precludes other substrates from entering the active cavity, decreases the heme redox potential, and thus slows down electron transfer from the redox partner CPR [57].

### 4.3. Inactivation of CYP3A through Heme Modification

A third mechanism for general CYP enzyme inactivation is heme modification such as heme destruction, mostly via the terminal alkyne, aldehyde, and furan functional groups present in various substrates. However, ritonavir does not contain any of these moieties, except for the isopropyl-thiazole ring, which can be cleaved and opened and may inactivate CYP3A4 via heme modification. The inactivation of CYP3A4 by heme destruction, along with the formation of a heme-protein adduct, was proposed and experimentally supported by Lin et al. (2013) [63]. These authors first confirmed that ritonavir is a potent mechanism-based inactivator of CYP3A4 due to heme destruction, without modification of the CYP3A apoprotein, using a reconstituted system containing *E. coli*-expressed CYP3A4 and CPR but not Cyt*b*_5_ [63]. They observed in their HPLC (high-performance liquid chromatography) analysis that after incubation with ritonavir plus NADPH, chromatographically detectable native heme was reduced by ~50%, whereas an apparent heme-protein adduct co-eluting with the CYP3A4 apoprotein was detected after the inactivation of CYP3A4 [63]. Cyt*b*_5_ was not added to this in vitro experimental system as its heme group would have interfered with the proper detection of the CYP3A4-derived heme moiety. Furthermore, spectral analysis of the suspected heme-protein complex suggested that heme was directly linked to the apoprotein of CYP3A4. In contrast, ritonavir was not found to be detectably linked to the apoprotein. The authors interpreted these results as most likely representing covalent cross-linking of the activated heme to the CYP3A4 apoprotein during the mechanism-based inactivation of CYP3A4 by ritonavir [63]. These results contrast with the results of Rock et al. discussed below, which were more favorable for the formation of a covalent adduct of ritonavir directly to the apoprotein of CYP3A4 without the inclusion of a heme residue [19].

**Figure 5:**
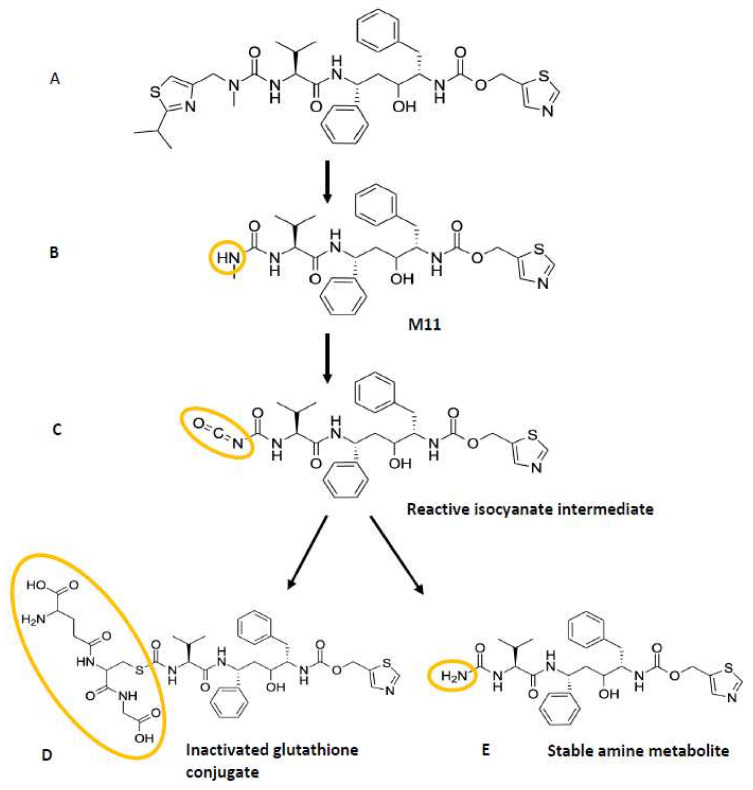
The proposed biotransformation scheme of the reactive isocyanate intermediate after conversion of ritonavir and further metabolism of its metabolite M11. The reactive isocyanate metabolite could be inactivated by forming a complex with GSH or could be further hydrolyzed into its stable amine metabolite. Chemical structures of (**A**) ritonavir, (**B**) M11 metabolite of ritonavir formed by loss of the isopropyl-methyl thiazole residue, (**C**) reactive isocyanate intermediate formed from the stable N-dealkylated metabolite, which has lost the HN-CH_3_ moiety, (**D**) an inactivated complex of GSH and the proposed reactive intermediate, and (**E**) stable amine metabolite formed after hydrolysis and loss of the -CO_2_ moiety of the reactive intermediate. The proposed reactive intermediate might initiate chemical activation of the heme group, leading to its ultimate conjugation to the CYP3A4 polypeptide [63]. The altered moieties of ritonavir in the various metabolites are highlighted in yellow.

Lin et al. further showed that glutathione (GSH) trapping of possible reactive intermediates formed by CYP3A4 from ritonavir yielded a complex of GSH and a ritonavir derivative that had lost the isopropyl-methyl thiazole residue. This proposed reactive intermediate might be an isocyanate intermediate of the stable ritonavir metabolite (M11) that was detected in the reaction mixture, formed by further loss of the HN-CH_3_ moiety of the metabolite (Figure 5). This proposed reactive isocyanate intermediate could form a complex with GSH or could be further hydrolyzed into a stable amine metabolite. Through the formation of a GSH conjugate, the proposed reactive intermediate will be inactivated. Perhaps this or similar reactive intermediates of ritonavir are responsible for initiating the chemical activation of heme leading to its conjugation to the CYP3A4 apoprotein. However, Lin et al. were not able to identify a dissociable heme adduct or an apoprotein adduct in their LC-MS/MS analysis, and, therefore, the mass of the purported reactive intermediate could not be elucidated [63]. The exact molecular structure of the reactive intermediate as suggested by this research group is, therefore, uncertain. This means that the contribution of this or perhaps of another reactive intermediate(s) to the mechanism-based inactivation of CYP3A4 is still not resolved.

### 4.4. Inactivation of CYP3A by Putative Covalent Linkage of a Reactive Intermediate to the CYP3A4 Apoprotein

A fourth mechanism of inactivation of CYP3A, again through the formation of a reactive ritonavir intermediate, was described by Rock et al. (2014) [19]. Koudriakova et al. (1998) were the first to suggest that there is the formation of a reactive intermediate between CYP3A and ritonavir, based on the observed mechanism-based inactivation of CYP3A in human liver and enterocyte microsomes, but without providing direct evidence for its existence [25]. Both recombinant-expressed CYP3A4 and CYP3A5 could metabolize ritonavir, yielding similar metabolite profiles and kinetic parameters. The biotransformation rates of ritonavir by human enterocyte and liver microsomes and recombinant-expressed CYP3A4 were found to be nonlinear as they decreased over time [25]. The observed nonlinearity and slowing down of the biotransformation of ritonavir were then shown to be directly related to the metabolism of ritonavir. The addition of the supernatant containing the ritonavir metabolites to a secondary incubation had no impact on the initial biotransformation velocity of ritonavir by intact microsomes. However, the metabolism rate did decrease quickly over time, even when enterocyte or liver microsomes were incubated with a large excess of ritonavir (<5% substrate disappearance over 1 h). These findings excluded the possibility of competitive inhibition by the generated ritonavir metabolites as an explanation for the nonlinearity [25]. Koudriakova et al. also preincubated HLMs with ritonavir and NADPH, and even after the removal of the drug by extensive washing, there was still a significant decrease observed in CYP3A metabolic activity. A 20 min preincubation of CYP3A-containing enterocyte microsomes with ritonavir and NADPH resulted in virtually complete inhibition of the metabolism of the CYP3A substrate indinavir, although ritonavir had been completely metabolized, and the resulting ritonavir metabolite mixture did not show significant inhibitory activity towards CYP3A. Therefore, these early data indicated that the metabolism of ritonavir is accompanied by the irreversible inactivation of CYP3A [25].

The inactivation of CYP3A by ritonavir in HLMs was also selective for CYP3A as there was no change observed in the formation of the hydroxylated isopropyl-thiazole ritonavir metabolite primarily formed by hepatic CYP2D6. Koudriakova et al. further considered the nature of the CYP3A4 inhibition by ritonavir and hypothesized that there may be the formation of a chemically reactive intermediate, possibly a fragment containing the isopropyl-methyl thiazole group, which would allow ritonavir to inactivate CYP3A [25]. This description fits the *N*-ritonavir (M1) structure, amongst others identified by Rock et al. as a CYP3A4 inactivator, as described below. However, this early study by Koudriakova et al. did not provide an explanation for how this putative reactive intermediate would then inactivate CYP3A4, for instance, by the binding of the reactive intermediate to the apoprotein or heme moiety.

The study of Rock et al. (2014) aimed to probe all the previously discussed mechanisms of CYP3A4 inhibition/inactivation. They reported that both ritonavir and one of its four main metabolites, *N*-ritonavir (M1) can efficiently inactivate CYP3A4, although the kinetics for *N*-ritonavir-mediated inactivation were slightly less efficient [19]. *N*-ritonavir is formed by CYP3A4-mediated cleaving off the methyl-ethyl thiazole group (deacylation), leaving a primary amine (Figure 3) [19]. This primary amine could in theory be involved in MIC formation via further metabolic activation, which was also earlier suggested by Ernest et al. [19,53]. Interestingly, in an earlier work by Sevrioukova et al. (2010), ritonavir had shown an enhanced CYP3A inhibitory effect after preincubation with HLMs, which together with its kinetic behavior suggested that ritonavir is a mechanism-based inhibitor because this was a time-dependent process. It might also suggest that a ritonavir metabolite (such as perhaps M1) is primarily responsible for this inhibition process [61].

In contrast to the earlier study of Ernest et al. (2005), Rock et al. did not observe detectable MIC formation with ritonavir in either CYP3A4 supersomes or HLMs (both supplemented with Cyt*b*_5_ and CPR) based on spectrophotometric absorbance spectra analysis. However, upon direct incubation with *N*-ritonavir, there was a MIC formed for nearly 100% of the CYP3A4 present, as judged by the spectrophotometric changes. Nonetheless, the concomitant addition of increasing concentrations of ritonavir very efficiently competed with this apparent MIC formation by *N*-ritonavir. It was also found that the ritonavir-inhibited CYP3A4 activity could not be restored by forced oxidation with ferricyanide, which is different from the behavior of a normal MIC, as was observed with CYP3A4 and the MIC-positive control troleandomycin. In contrast, for *N*-ritonavir-inhibited CYP3A4, this restoration was possible, further supporting a normal MIC formed by the latter compound but not by ritonavir [19].

In other words according to this study [19], although one of the primary ritonavir metabolites formed by CYP3A4 (*N*-ritonavir, M1) can in principle act as an efficient MIC-type inhibitor of CYP3A4, this does not play a quantitatively significant role for ritonavir. This suggests that upon first formation from ritonavir in the CYP3A4 enzyme active site, *N*-ritonavir is not in a position or configuration to allow MIC formation with CYP3A4, as otherwise this metabolite would not normally be released from the enzyme but instead proceed to form a MIC. One possibility is that the cleaved-off thiazole moiety still residing in the active site physically prohibits the *N*-ritonavir from assuming a position necessary to form a MIC with the heme moiety within the active site of CYP3A4. Perhaps only after the release of both metabolic products from the active site and rebinding of *N*-ritonavir, a MIC can be established. The data also suggest that ritonavir, once tightly bound, efficiently blocks the re-entry of previously formed and released *N*-ritonavir into the enzyme, otherwise, over time a gradual increase in MIC formation (with *N*-ritonavir) would be expected upon incubation with ritonavir alone [19]. Conversely, the competition data also suggested that MIC formation with *N*-ritonavir blocks the subsequent entry of ritonavir (this is in contrast to the situation with concomitant exposure). These findings suggested that the metabolism of ritonavir by CYP3A4 does not directly lead to an inactivating MIC, for instance, through the rapid formation of *N*-ritonavir [19], but it may do so indirectly by the release and subsequent rebinding of *N*-ritonavir.

Rock et al. then hypothesized that ritonavir could be metabolized to a reactive intermediate that is capable of forming a covalent bond to the prosthetic heme of CYP3A4, thus inactivating CYP3A4, as was earlier suggested by Lin et al. (2013) [19,63]. Usually, the covalent (alkylating) modification of the heme group in CYP3A would result in an altered CO differential spectrum upon CO binding to the heme group. To test this hypothesis, they performed a differential CO-binding spectrum experiment with reconstituted CYP3A4, containing purified CYP3A4, CPR, and Cyt*b*_5_ in a 1:1:2 molar ratio. This experiment yielded no indication that there was a heme adduct formed during the NADPH-dependent enzyme inactivation by ritonavir. Moreover, co-incubated radioactive ritonavir was not found to be covalently bound to the heme group after the denaturation of the protein [19].

As the inactivation of CYP3A4 by ritonavir could not be explained by either MIC formation or heme modification in their analysis, Rock et al. (2014) further studied whether ritonavir might be covalently linked to the polypeptide (apoprotein) fraction of CYP3A4. On both CYP3A4 supersomes and HLM incubations, radioactive ^3^H-ritonavir was found to bind to the subsequently denatured (and thus presumably heme-free) and extensively washed protein fractions in an NADPH-dependent way. The measured molar ratio of the bound radiolabel equivalents and CYP3A4 protein in the precipitate fractions was close to 1 (0.87–0.91) [19]. This process was further investigated using reconstituted CYP3A4 containing purified CYP3A4, rat CPR, and rat Cyt*b*_5_, and incubation with a 15-fold molar excess of radioactive ritonavir at plus or minus NADPH. Liquid chromatography analysis of the denatured reaction mixture yielded separate heme and polypeptide peaks at their expected elution positions, with radioactive ritonavir co-eluting primarily with the CYP3A4 polypeptide in the plus NADPH reaction and not with heme. Again, the calculated bound radiolabel equivalent to the (injected) CYP3A4 apoprotein molar ratio was close to 1 (0.91) [19]. These data suggested the formation of a nearly equimolar conjugation complex between ritonavir and the CYP3A4 apoprotein. It should, however, be noted that quantitative assessments based on detected radioactivity can be confounded unless it is certified that all detected radioactivity is residing in the conjugated ritonavir.

A subsequent similar incubation of reconstituted CYP3A4 with a 20-fold molar excess of ritonavir or deuterated ritonavir-*d*_6_, followed by whole-protein mass spectrometry revealed, in addition to an abundant CYP3A4 apoprotein peak at 56,259 Da, a considerably lower peak that was 737 (with ritonavir incubation) or 743 Da (with ritonavir-*d*_6_ incubation), respectively, suggesting the addition of one entire ritonavir molecule plus 16 a.m.u., likely representing an O-atom, to a fraction of the apoprotein. Again, the emergence of this higher molecular-weight peak was NADPH-dependent. Tryptic and proteinase K digestions of the resulting product showed that (radioactive) ritonavir was bound to only a single specific CYP3A4 peptide sequence (255-RMKESRLEDTQKHR-268). In addition, the stoichiometry between the peptide-bound radioactive ritonavir and CYP3A4 input was calculated to be close to one (0.87), suggesting that nearly every CYP3A4 apoprotein molecule had acquired a ritonavir adduct [19]. It should be noted, however, that the original whole-protein mass spectrometry experiment had revealed a much more abundant apoprotein peak than the assumed apoprotein plus ritonavir peak, indicating only a very partial ligation of ritonavir to the apoprotein. No explanation for this apparent discrepancy with the earlier smaller-scale results was given by the authors [19].

Further analysis of both the tryptic and proteinase K digests of the separated incubation product showed that ritonavir was bound to lysine residue 257 (Lys257) in CYP3A4 and that the isopropyl-thiazole end of the ritonavir molecule was retained in the protein adduct and, therefore, likely closest to the apoprotein. Interestingly, it has been shown that ritonavir requires the presence of both thiazole rings to exhibit its potent inhibitory effects [19]. Unfortunately, the exact structure of the moiety adducted to Lys257 was not resolved. However, the authors suggest that a reactive sulfoxide may have been formed by CYP3A4 on the isopropyl-thiazole ring (Figure 1), followed by a subsequent nucleophilic attack on the ring by the terminal amine of Lys257 [19]. This reactive intermediate thus formed a covalent bond to Lys257 of the CYP3A4 apoprotein [19].

CYP3A4 contains many lysine residues, and the unique ligation of ritonavir to Lys257 led the authors to hypothesize that Lys257 may be located in an egress channel of CYP3A4 for its substrates. Altogether, these authors concluded that the irreversible inactivation of CYP3A4 upon the metabolism of ritonavir occurs primarily by covalent attachment of an oxidized ritonavir fragment (reactive intermediate) to Lys257 of the CYP3A4 apoprotein [19]. The assessment of this being the primary mechanism was largely based on the observed stoichiometry of close to one for the bound ritonavir radiolabel equivalents and CYP3A4 apoprotein or peptide. An interesting prediction of this model is that some ritonavir analogues that are modified around the isopropyl-thiazole residue should no longer be mechanism-based inhibitors of CYP3A4.

## 5. Discussion and Conclusions

**Figure 6:**
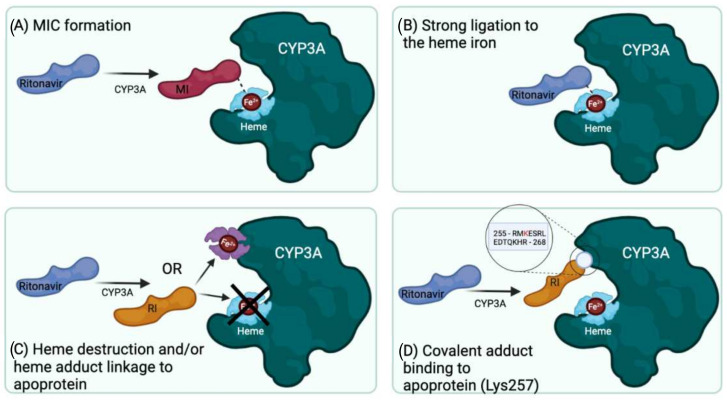
Highly schematic overview of the four main mechanisms of inactivation of CYP3A4/5 by ritonavir proposed in the reviewed studies. For simplicity, CYP3A is depicted here in a wide-open static configuration and no attempt was made to mimic real 3D structures. MI, metabolic intermediate; RI, reactive intermediate.

Based on current knowledge and literature, it remains difficult to arrive at a definitive conclusion on the actual primary mechanism of the (quasi-) irreversible inactivation of CYP3A by ritonavir. The reviewed studies suggest at least four fundamentally different mechanisms (Figure 6): (A) quasi-irreversible MIC formation; (B) extremely tight binding of unchanged ritonavir to the heme iron; (C) heme destruction followed by heme-protein adduct formation; and (D) formation of a reactive ritonavir intermediate, which attaches to the apoprotein. A further complication is that ritonavir is also metabolized by CYP3A, and the formed metabolites could inhibit the enzyme through covalent or non-covalent binding. Still, based on the reviewed studies, we can conclude that ritonavir is a mechanism-based inactivator/inhibitor, without arriving at a definitive answer as to the primary mechanism of action. As far as we can judge, all of the reviewed studies appear to be well designed and executed, with only minor comments on the experimental setups and interpretations, which will be discussed below. This begs the question of what is then the correct explanation for the potent inhibition of CYP3A by ritonavir? Based on current knowledge, we think that we should leave the possibility open that ritonavir significantly inactivates CYP3A in multiple ways, thus resulting in a mixed mechanism of (quasi-) irreversible inhibition. This probably depends on the way ritonavir approaches and binds in the enzyme active site, determining whether a MIC or reactive intermediate is formed. Matters become even more complicated since some of the known CYP3A metabolites of ritonavir, such as *N*-ritonavir, can also form MICs with CYP3A4. Therefore, it may not always be possible to discriminate the effects of ritonavir from those of its metabolites.

Looking into the experimental designs of the reviewed studies, we note that a potentially important difference between them is the absence or presence of Cyt*b*_5_ in the in vitro assay systems (see Table 1), which may have had an impact on the behavior of CYP3A4 with respect to ritonavir. In addition to NADPH and CPR, Cyt*b*_5_ can also modulate electron donor functions towards P450 enzymes, including CYP3A4. Cyt*b*_5_ likely also plays an important role in vivo for human CYP3A4-mediated drug metabolism. This was suggested by the studies of Henderson et al., who showed that in transgenic mice overexpressing human CYP3A4, the hepatic deletion of (mouse) Cyt*b*_5_ resulted in a markedly reduced in vivo metabolic activity of CYP3A4 [71]. In this respect, it should be noted that the studies of Sevrioukova et al. (from 2010) and Lin et al. (2013) performed most biochemical experiments in the absence of Cyt*b*_5_, which may have affected some of their outcomes. In contrast, Rock et al. (2014) did include Cyt*b*_5_ in most experiments, which may make their results more representative of the in vivo situation. It is worth noting that in the experiments of Ernest et al. (2005), Cyt*b*_5_ was not present in the tested CYP3A5 preparation, whereas it was present in the tested CYP3A4(+*b*_5_) preparation. This is despite the fact that it has been demonstrated that Cyt*b*_5_ markedly increases the substrate metabolism rates of both CYP3A4 and CYP3A5 [72]. This raises the question of whether factors other than just CYP3A4 or -3A5 and ritonavir (plus CPR and NADPH) could affect the extent and rate of MIC formation or, indeed, the primary inactivation mechanism of CYP3A in the reviewed experiments.

It remains unclear why Ernest et al. could spectrophotometrically detect MIC formation upon the incubation of ritonavir and CYP3A4, whereas Rock et al. could not [19,53]. However, there were subtle differences in the sources and reconstitution mixtures of the enzymes used (e.g., differences in protein tags of CYP3A4 and the species origin and relative molar ratios of Cyt*b*_5_ and CPR used relative to CYP3A) that could not always be unambiguously reconstrued using the described methods. Furthermore, Ernest and co-workers did not report a structure of the suggested MIC, the existence of which was only based on the observed spectrophotometric absorbance shift around 455 nm. This makes it difficult to prove that MIC formation is the actual primary inhibitory mechanism of CYP3A4 by ritonavir. Unlike CYP3A4 (+Cyt*b*_5_), no MIC formation could be spectrophotometrically detected when ritonavir was incubated with HLMs or CYP3A5 (not supplemented). However, CYP3A5 was also irreversibly inhibited by ritonavir—albeit 3–4 times more slowly than CYP3A4. This could, therefore, have been a matter of detection sensitivity. Moreover, the suggested MIC formation by Ernest et al. only accounted for 64% of the total inactivated CYP3A4 so this may not be the only mechanism of inhibition by ritonavir in these studies [19,53]. These results further support the possibility of a mixed inactivation mechanism of CYP3A by ritonavir of which the individual contribution to inhibition is probably not equally divided by the four mechanisms. Therefore, it might be helpful to perform further experiments on possible MIC formation to elucidate this. Of note, in a study by Ernest and co-workers, MIC formation was only (spectrophotometrically) studied using HLMs. This raises the question of whether the preincubation of ritonavir with HLMs is the best way to verify MIC formation as a possible inactivation mechanism of CYP3A. HLMs contain other CYP enzymes besides CYP3A4 or -3A5, which could interact with ritonavir. Therefore, HLMs are not optimal for studying MIC formation, and thus the use of CYP3A4 supersomes might be more suitable for evaluating MIC formation by CYP3A4.

As mentioned above, the X-ray structural binding experiments of Sevrioukova and co-workers were mostly conducted in the absence of the natural reducing partners of CYP3A4, CPR, or Cyt*b*_5_, which may well have limited the number of possible outcomes of their experiments. Their initial studies suggested that both thiazole rings are needed for the strong CYP3A inhibitory potency of ritonavir [19,70]. Currently, ritonavir is still the most potent CYP3A4 inhibitor, but in more recent studies, Sevrioukova et al. investigated structurally related molecules to possibly obtain an even more potent inhibitor [56,73]. An important implication of their studies was that ritonavir could approach CYP3A4 in different orientations, which could allow the formation of different reactive intermediates and metabolites. The ones that are formed would be highly dependent on the way ritonavir enters the active site cavity. In addition to their proposed primary inactivation mechanism, Sevrioukova et al. suggested the possibility of the separate formation of a reactive intermediate from ritonavir, which also strongly ligates to the heme iron of CYP3A4. Clearly, these authors did not exclude other events from occurring in addition to their favored mechanism of irreversible inhibition. Some of these may be represented by the other basic inactivation mechanisms discussed in this review. More generally speaking, the observation that CYP3A4 can generate a number of different ritonavir metabolites directly proves that different fates of ritonavir arise on its binding to CYP3A4 and that effective inhibition does not always occur instantly. What applies to the ritonavir metabolizing events could also apply to the ritonavir irreversible inhibition events.

**Figure 7:**
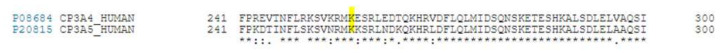
Alignment of the amino acid sequences of human CYP3A4 (top) and CYP3A5 (bottom) from position 241 to 300. The Lys257 (K257) residue is highlighted. *, amino acids are identical; conservative substitution; and, non-conservative substitution between both enzymes. P number in blue: protein entry identification code.

In striking contrast, the experiments of Rock et al. appeared to rule out the suggested MIC formation and the heme modification or destruction pathways for ritonavir. Instead, they documented the formation of a reactive isopropyl-thiazole intermediate, which attaches to the peripheral Lys257 residue of the apoprotein of CYP3A4, providing sequence evidence for this adduct [19]. It is worth noting that an interesting way of directly addressing whether Lys257 is indeed essential for the mechanism of irreversible inhibition of CYP3A4 by ritonavir would be to mutate this residue to some other amino acid(s) that is unlikely to react with the activated ritonavir metabolites to see whether the irreversible inactivation still occurs. Of course, this approach would only work if these mutant(s) maintained most of the properties of CYP3A4 in metabolizing ritonavir. It may further be interesting to consider if this proposed mechanism could be translated to the ritonavir-CYP3A5 inactivation since ritonavir appears to be almost as effective in the “mechanism-based” inactivation of CYP3A5. Comparing the amino acid sequences of both enzymes (Figure 7), CYP3A5 exhibits a lysine residue at the analogous position 257, which could be involved in a similar way to the suggested mechanism for CYP3A4. Interestingly, the surrounding sequence is also quite highly conserved, except for the neighboring position 258, which consists of CYP3A4 of the negatively charged glutamic acid in contrast to the positively charged lysine residue in CYP3A5.

Even after reviewing the current insights into the proposed mechanisms of CYP3A inactivation, the question remains of why ritonavir is such an effective irreversible inhibitor of CYP3A4 and still one of the most effective CYP3A-inhibiting compounds around. Perhaps the possibility of a number of different inactivation routes occurring in parallel between ritonavir and CYP3A4, as suggested by the various reviewed studies, can partly explain this efficacy. This may relate to the combination of the promiscuity of CYP3A4, allowing many different ways to accommodate/bind ritonavir in its active site, and the relative complexity, flexibility, and multi-functionality of the ritonavir molecule. This allows many different moieties of the molecule to be metabolized, activated, or tightly bonded and could be behind the presumed multitude of possible inactivation routes open to this complex. This could simply be dependent on the different configurations in which ritonavir is initially lodged in the active site of CYP3A4. In turn, this flexibility could give rise to a range of different pathways along which the complex can move after initial binding. It may further be interesting to consider how the suggested mechanisms in this review, which explain the ritonavir-CYP3A4 inactivation, translate to ritonavir-CYP3A5 inactivation. After all, ritonavir appears to be almost as effective in the “mechanism-based” inactivation of CYP3A5, and one would expect similar mechanisms to play a role for these closely related proteins. We note that a systematic, side-by-side experimental analysis of the biochemical mechanism of the irreversible inhibition of CYP3A4 and CYP3A5 by ritonavir could shed some more light on the most likely primary mechanism of inactivation of ritonavir of these two proteins. For example, spectral binding experiments and inactivation assays of CYP3A4 and -3A5 using identically supplemented supersomes of both enzymes could possibly elucidate such answers.

In summary, it is surprising that at least four fundamentally different mechanisms still need to be considered as the potential main way in which ritonavir irreversibly inactivates CYP3A. Based on the apparent quality of the reviewed studies, it seems unlikely that any of the proposed mechanisms are completely wrong. In fact, from each of the proposed studies and mechanisms, it is hard to conclusively exclude that any of the alternative mechanisms (also) play a significant role. It, therefore, seems most likely that ritonavir can exhibit multiple mechanisms of the irreversible inactivation and inhibition of CYP3A. However, which one of these (if any) is predominant in the human clinical situation remains open to debate. Perhaps all four mechanisms discussed here can significantly contribute to the inactivation of CYP3A. If so, this might help to explain the overall efficacy of the inactivation process. Importantly, despite the complex chemical structure of ritonavir and the apparent differences in binding to CYP3A4 and CYP3A5, there is no indication that either protein is inhibited more strongly than the other by ritonavir. Therefore, independent of the exact inhibitory mechanism, ritonavir is able to irreversibly inactivate CYP3A4 and -3A5 to more or less the same extent. This is obviously a major advantage for the use of ritonavir as a clinical booster because the boosting effect can then be expected to be quite similar in various ethnic and mixed populations. However, it is clear that the final word is yet to come on the primary mechanism by which ritonavir irreversibly inhibits CYP3A.

## Figures and Tables

**Table 1:** Systematic overview of the divergent proposed mechanisms of mechanism-based inactivation of CYP3A by ritonavir and the used experimental approaches.

Reference	Suggested Primary Mechanism of Inactivation	Used Enzyme Preparations	Use of Added Cyt*b*_5_ or CPR in the Experiments *	Incubation Time	Assay(s)	Ritonavir Concentration
Koudriakova et al. (1998) [25]	Reactive intermediate formation	Enterocyte microsomes and HLMs expressing CYP3A4, -3A5, and -2D6	- No Cyt*b*_5_ - No CPR	1 h	Time-course assay using HPLC to examine the rate of ritonavir metabolism	2 or 5 µM
				20 min	Inactivation of CYP enzymes assay using HPLC	0.075 µM
Ernest et al. (2005) [53]	MIC formation	HLMs expressing CYP3A4 and -3A5	- Recombinant CYP3A4: with Cyt*b*_5_ - Recombinant CYP3A5: no Cyt*b*_5_ - All with CPR	Maximally 60 min	CYP3A4/5 inactivation and high-affinity binding assay with testosterone substrate to quantify time- and concentration-dependent loss of CYP3A activity	0.05, 0.10, 0.20, 0.50, and 1 µM
Sevrioukova et al. (2010) [61]	Strong ligation of ritonavir to heme iron	Isolated CYP3A4Δ3-24	- No Cyt*b*_5_ - No CPR	-	Kinetic assay of CYP3A4-ritonavir binding using stopped-flow spectrophotometry to measure the kinetics of ritonavir binding to ferric and ferrous P450 by monitoring absorbance changes at 426 and 442 nm	0.5–30 μM
					Crystallization and structure determination with bound ritonavir	Ritonavir-bound CYP3A4 protein (50–60 mg/mL)
Lin et al. (2013) [63]	Heme destruction and linkage of heme to apoprotein	Purified CYP3A4 and CYP2B6, and HLMs	- No Cyt*b*_5_ - With CPR	30 min	Enzyme and inactivation assay of CYP3A4 and CYP2B6 to determine catalytic activity using a fluorescence plate reader	0.5–20 µM
				10 min	HPLC analysis of heme iron to study the loss of native heme and formation of heme adducts	10 µM for CYP2B6; 2 µM for CYP3A4
				10 min	ESI–LC/MS analysis of the apoprotein to study the mass spectra	10 µM
				20 min	LC-MS/MS analysis of ritonavir metabolites and the GSH conjugate formed	40 µM
Rock et al. (2014) [19]	Reactive intermediate formation with covalent adduct binding to apoprotein (Lys257)	CYP3A4 supersomes or HLMs	- With Cyt*b*_5_ - With CPR	30 min, after 3 min pre-incubation	CYP3A4 activity and inactivation assay using midazolam with a UPLC system and LC-MS/MS for the inactivation assay	0–10 µM of ritonavir or *N*-ritonavir
					MIC formation assay using spectrophotometric repetitive scanning from 430–495 nm over 30 min	10 µM
			With and without NADPH	10 min	Mass spectral analysis of CYP3A4 peptides using a liquid chromatography - radioisotope counting system	10 µM

* This column only contains the information of the experiments that were essential for the conclusions of the authors regarding their proposed mechanism of inactivation of CYP3A by ritonavir. In fact, the reviewed studies showed more detailed information including the experimental conditions. HLMs, human liver microsomes. CYP, cytochrome P450. Cyt*b*_5_, cytochrome b5. CPR, cytochrome P450 reductase. NADPH, nicotinamide adenine dinucleotide phosphate. MIC, metabolic intermediate complex. GSH, glutathione. Lys257, lysine 257. HPLC, high-performance liquid chromatography. ESI-LC/MS, electrospray ionization mass spectrometry. LC-MS/MS, liquid chromatography with tandem mass spectrometry. UPLC, ultra-high-performance liquid chromatography.

## Data Availability

The data presented in this study are openly available in the reviewed studies published online and accessible through PubMed.
